# Rivastigmine in moderately severe-to-severe Alzheimer’s disease: Severe Impairment Battery factor analysis

**DOI:** 10.1186/alzrt229

**Published:** 2013-12-18

**Authors:** Steven Ferris, Stella Karantzoulis, Monique Somogyi, Xiangyi Meng

**Affiliations:** 1 Alzheimer’s Disease Center, Comprehensive Center on Brain Aging, NYU Langone Medical Center, Room 506, 145 East 32nd Street, New York, NY 10016, USA; 2 Novartis Pharmaceuticals Corporation, East Hanover, NJ 07936-1080, USA

## Abstract

**Introduction:**

The Severe Impairment Battery (SIB) is validated for assessing cognition in patients with severe dementia. The current analysis aimed to further investigate the cognitive efficacy of rivastigmine capsules, as assessed by SIB factor scores, in patients with moderately severe-to-severe Alzheimer’s disease (AD).

**Methods:**

This was a retrospective analysis of a 26-week, multicenter, randomized, double-blind, placebo-controlled study of oral rivastigmine conducted in Spain. Previously reported outcome measures included the full SIB. Current analyses examined calculated scores and effect sizes for the change from baseline at Week 26 on: newly defined SIB subscales (derived by a factor analysis of the 40 SIB items, using the PROC FACTOR function (SAS)); previously defined memory, language and praxis subscales (derived by previous analysis of the nine SIB domains); and the individual SIB items. Treatment differences were assessed.

**Results:**

SIB data were provided by 104 rivastigmine-treated patients and 106 patients receiving placebo (Intent-To-Treat Last Observation Carried Forward population). Significantly less decline was observed on the previously defined memory and language subscales, and the newly defined working memory/memory subscale in rivastigmine-treated patients (all *P* < 0.05 versus placebo). Calculation of effect sizes demonstrated numerically greater efficacy of rivastigmine versus placebo on each of the subscales, and a broad range of SIB items; greatest effect sizes were observed on SIB items assessing the current month (effect size = 0.30) and digit span series (effect size = 0.33).

**Conclusions:**

These data suggest the observed efficacy of rivastigmine in moderately severe-to-severe AD is likely a cumulative effect across a range of tasks. Rivastigmine demonstrates broad cognitive efficacy in this patient population.

## Introduction

The cholinesterase inhibitor rivastigmine has demonstrated efficacy on activities of daily living (ADL), cognition and behavior in clinical trials of patients with mild-to-moderately severe Alzheimer’s disease (AD) [1-4]. Currently, oral (6 to 12 mg/day) and transdermal rivastigmine (4.6 mg/24 h, 9.5 mg/24 h and 13.3 mg/24 h) are approved in the USA for the symptomatic treatment of mild-to-moderate AD [5,6]. A previously reported pooled analysis of data from clinical trials investigating the efficacy of rivastigmine in patients with mild-to-moderately severe AD suggested that rivastigmine may also benefit patients at more advanced stages of the disease [7]. Indeed, the rivastigmine transdermal patch has recently been approved in the USA for the symptomatic treatment of severe AD [5]. This approval was based on the findings of the *ACT*ivities of daily living and cognit*ION* (ACTION) study, a large, 24-week, multicenter, randomized, active-controlled trial assessing the comparative efficacy and safety of 13.3 mg/24 h versus 4.6 mg/24 h rivastigmine transdermal patch in patients with severe AD [8].

The efficacy and safety of oral rivastigmine in Spanish patients with moderately severe-to-severe AD (Mini-Mental State Examination (MMSE) score of 5 to 12) were previously investigated in a 26-week, multicenter, randomized, double-blind, placebo-controlled trial [9]. In this study, rivastigmine treatment was associated with significantly less decline on the Severe Impairment Battery (SIB) and significant improvements on the MMSE and Alzheimer’s Disease Cooperative Study-Clinical Global Impression of Change (ADCS-CGIC) scale, compared with placebo [9]. A trend towards improvement, or reduced decline, was also seen in rivastigmine-treated patients compared with placebo on the 10-item Neuropsychiatric Inventory (NPI-10), the 4-item NPI (NPI-4), the Global Deterioration Scale (GDS) and the Alzheimer’s Disease Cooperative Study-Activities of Daily Living (ADCS-ADL) scale [9]. Of the assessment scales used in this study, the SIB was specifically designed and validated for use in patients with severe dementia, since they may have difficulty completing more challenging standard neuropsychological assessments, [10-12] such as the Alzheimer’s Disease Assessment Scale-cognitive subscale (ADAS-cog).

The SIB is a performance-based measure that assesses nine symptom domains: attention, language, orientation, memory, praxis, visuospatial perception, construction, social skills and orientating head to name [10-12]. Several variations of this test have been developed, with a range of possible total scores [10-12]. One such version of the SIB assesses patients on 40 items (questions) to give a total score ranging from 0 to 100, where a lower score is indicative of more severe impairment [11].

Previous analyses have grouped the nine domains of the SIB into three higher-order subscales: memory, language and praxis (Table 1) [13]. However, the specific cognitive functions comprising these higher-order subscales are broad; for example, the language domain contains items that assess more than just language (for example, naming the months of the year, which could also be considered a memory assessment). Wider domain coverage is also true for the memory and praxis subscales. Redefining the subscales of the SIB using a new factor analysis of the 40 individual SIB items, as opposed to the broad SIB domains, as well as examining treatment effects on individual items, may facilitate further investigation of the efficacy of rivastigmine on more specific aspects of cognition in patients with severe AD.

**Table 1:** **Previous organization of the SIB domains into higher-order subscales**[13]

**Higher-order subscale**	**SIB domain**
Memory	Memory
	Attention
	Orientation
	Orientating head to name
Language	Language
	Social skills
Praxis	Praxis
	Construction
	Visuospatial perception

SIB, Severe Impairment Battery.

The aim of the current *post-hoc* analysis was to further investigate the efficacy of oral rivastigmine on cognition in severe AD, using SIB item data from the previously reported clinical trial of oral rivastigmine in moderately severe-to-severe AD [9]. More specifically, the subscales of the SIB were redefined by performing a factor analysis of the individual items, as opposed to starting with the previously defined symptom domains. We investigated the pattern of efficacy of rivastigmine 1) on these newly defined subscales; 2) on the memory, language and praxis higher-order subscales of the SIB derived by a previous analysis [13]; and 3) for each of the individual SIB items.

## Methods

This was a *post-hoc* analysis of a 26-week, multicenter, randomized, double-blind, placebo-controlled, parallel-group study conducted in Spain that evaluated oral rivastigmine treatment in patients with moderately severe-to-severe AD [9]. The full details of the design and conduct of this study have been published previously [9]. Briefly, patients were male or female, aged 55 years or older, with probable AD (Diagnostic and Statistical Manual of Mental Disorders, 4^th^ edition and the National Institute of Neurological and Communicative Disorders and Stroke-Alzheimer’s Disease and Related Disorders Association criteria) of moderately severe-to-severe intensity (MMSE score 5 to 12, GDS stage 5 to 6) [9]. Patients randomized to rivastigmine were up-titrated to the target dose of 6 mg rivastigmine capsules twice-daily (bid) in four-week steps (using 1.5 mg bid, 3 mg bid and 4.5 mg bid as intermediate doses) [9]. This titration schedule was modified in the event of any tolerability issues. Outcome measures included the change from baseline at Week 26 on the SIB [9]. This study received ethical committee approval and was conducted according to the ethical principles of the Declaration of Helsinki, as revised in 2000 [9].

Baseline SIB data from this study were used to perform a new factor analysis to establish a ‘best fit’ for each of the 40 individual SIB items into possible new subscales determined by the factor analysis. For this new factor analysis, the PROC FACTOR function in SAS was used. Initial common factor extraction was performed using the principal component method. Estimates of loadings were obtained using varimax rotation. Items were allocated to factors based on loadings and highest factor scores.

The least-squares (LS) mean change from baseline at Week 26 on the previously published (memory, language and praxis) [13] and newly defined subscales of the SIB were calculated for the Intent-To-Treat Last Observation Carried Forward (ITT-LOCF) population. Additional analyses were also performed based on the Observed Case (OC) population. For the ITT-LOCF approach, the statistical significance of treatment differences was assessed using an analysis of covariance (ANCOVA) model, with treatment and center as factors, and baseline as a covariate. For the ITT-OC approach, the statistical significance of treatment differences was assessed using a repeated measure ANCOVA model, with treatment, center, time-point and treatment-by-time point as factors, and baseline as a covariate. *P*-values were calculated from LS means.

Effect sizes (Cohen’s d) were calculated, based on mean values, to compare the change from baseline at Week 26 on the newly defined SIB subscales, the previously defined SIB subscales and each of the individual items of the SIB in patients randomized to receive rivastigmine or placebo.

## Results

### Patients

In the 26-week, multicenter, randomized, double-blind, placebo-controlled, parallel-group trial on which this analysis is based, 218 patients were randomized (1:1) to receive rivastigmine or placebo [9]. Five rivastigmine-treated patients and three patients in the placebo group did not provide a post-treatment SIB assessment and were excluded from the analyses. Therefore, the ITT-LOCF population comprised 210 patients (104 in the rivastigmine group, and 106 in the placebo group) [9]. The ITT-OC population at Week 26 comprised 194 patients (96 in the rivastigmine group and 98 in the placebo group). At Week 26, the mean (standard deviation (SD)) dose of rivastigmine received by the rivastigmine group was 9.8 (2.8) mg/day [9].

At baseline, both treatment groups were similar in terms of their baseline demographics and patient characteristics. The mean age of the rivastigmine-treated group and the placebo group was 78.0 (range 60 to 89) and 77.2 (range 57 to 92) years, respectively. Seventy-eight percent of the rivastigmine-treated patients and 76% of the placebo group were female. The mean (SD) MMSE score at baseline was 9.0 (2.2) in the rivastigmine group and 8.7 (2.2) in the placebo group, indicating moderately severe-to-severe impairment [9].

### Factor analyses

The new factor analysis performed on the SIB at baseline allocated each of the 40 individual items to one of five factors. Allocation was based on which factor was associated with the highest factor score for each individual item (Table 2). The individual items allocated to each factor, and their corresponding SIB domain, are summarized in Table 3. Based on the individual items comprising these factors and their clinical relevance, we named Factor 1, visual; Factor 2, language; Factor 3, working memory/memory; Factor 4, praxis and social skills; and Factor 5, naming.

**Table 2:** Factor scores derived by factor analysis to allocate the SIB items into new subscales

**SIB item**	**Factor 1: visual**	**Factor 2: language**	**Factor 3: working memory/memory**	**Factor 4: praxis and social skills**	**Factor 5: naming**
1a: Shake hands	0.18637	0.10742	0.14187	**0.46777**	0.06278
1b: Follow directions	0.03926	0.16075	0.09979	**0.72810**	−0.02260
1c: Sit/move to table/pull tray	0.14512	0.07164	0.03912	**0.81773**	0.09756
2: Examiner’s name (immediate recall)	0.19391	0.01890	**0.35278**	0.20451	0.00918
3: Subject’s name	0.13280	0.09591	0.11500	**0.27894**	0.13269
4a: Write name	0.14911	**0.83191**	0.21540	−0.01825	−0.13753
4b: Copy name	0.18811	**0.79331**	0.22121	0.02954	−0.17792
5: Current month	0.09048	0.10255	**0.16135**	−0.00452	0.08214
6: Months of the year	−0.08840	**0.44571**	0.16330	0.06817	0.13195
7: City	0.03403	0.23398	**0.47705**	0.03550	0.13178
8a + 8b: Descriptive naming (cup + spoon)	0.19170	0.11481	0.45178	0.17732	**0.53765**
9a: Reading comprehension	0.04419	**0.56384**	0.01299	0.09907	0.24333
9b: Verbal comprehension	0.10071	**0.58147**	0.04924	0.17725	0.22978
9c: Reading	0.06602	**0.75731**	−0.03219	0.10638	0.12338
10: Sentence recall	0.12520	**0.43543**	0.27214	0.11551	0.15668
11a + 11b: Repetition (people spend money + baby)	0.17676	0.15277	0.29899	**0.46208**	0.21274
12: Digit span (series)	−0.01152	0.12864	**0.39120**	0.09214	0.02801
13: Verbal fluency	0.22551	0.00574	**0.60501**	0.16787	0.25116
14: Examiner’s name (delayed recall)	0.02598	0.04244	**0.22845**	0.06847	−0.00027
15 + 20: Naming object in photograph (cup + spoon)	0.20978	0.21774	0.18377	0.15042	**0.67844**
16 + 18: Using cup (photograph + object)	0.24127	0.04208	**0.48144**	0.06123	0.41299
17 + 22: Naming object (cup + spoon)	0.19141	0.15694	0.16444	0.19876	**0.70735**
19 + 24: Forced choice naming (cup + spoon)	0.34707	0.04743	0.04278	0.33749	**0.37978**
21 + 23: Using spoon (photograph + object)	0.24747	0.06307	**0.40561**	0.15432	0.39211
25: Immediate recall of objects (cup + spoon)	0.30110	0.10494	**0.46109**	0.22645	0.17432
26 + 30a + 30b: Color naming (blue + red + green)	**0.52207**	0.21932	0.07852	0.03810	0.36658
27: Color matching	**0.59979**	0.05950	0.12284	0.12189	0.17387
28: Colored block	**0.56639**	0.10328	0.02037	0.12734	0.05381
29: Color discrimination	**0.68207**	0.13255	0.28264	0.15338	0.11077
31: Shape matching	**0.54832**	0.13483	0.23207	0.22148	0.32796
32: Shape	**0.55816**	−0.04496	0.22437	0.19948	0.09445
33: Shape discrimination	**0.58847**	0.06018	0.38125	0.26172	0.10459
30c + 34a + 34b: Shape identification (square + circle + triangle)	**0.36032**	0.26761	0.33625	0.21808	0.20124
35a: Drawing (circle)	0.23055	0.20295	**0.28592**	0.04922	0.25047
35b: Drawing (square)	0.23820	0.30433	**0.47077**	0.06087	0.13839
36: Auditory span	0.21216	0.12792	**0.28611**	0.27011	0.10342
37: Visual span	0.26459	0.28920	**0.30645**	0.17403	0.30167
38: Delayed recall of objects (cup + spoon)	0.37135	−0.04291	**0.47166**	0.16280	0.07484
39: Orientation to name	0.15592	0.01837	0.16938	**0.58642**	0.14839
40: Language ability (free conversation)	0.16147	−0.07097	0.40767	**0.47483**	0.16766

SIB, Severe Impairment Battery. Scores were derived using initial common factor extraction and the principal component method, and were based on data from 217 patients. Estimates of loadings were obtained using varimax rotation. SIB items were allocated to factors on the basis of highest score. Items allocated to each factor are shown in bold.

**Table 3:** Allocation of the individual items of the SIB into five new subscales

**New subscale**	**SIB item**	**SIB domain [**[10]**]**
Factor 1: visual	26 + 30a + 30b: Color naming (blue + red + green)	Language
	27: Color matching	Visuospatial perception
	28: Colored block	Memory
	29: Color discrimination	Visuospatial perception
	30c + 34a + 34b: Shape identification (square + circle + triangle)	Language
	31: Shape matching	Visuospatial perception
	32: Shape	Memory
	33: Shape discrimination	Visuospatial perception
Factor 2: language	4a: Write name	Language
	4b: Copy name	Language
	6: Months of the year	Language
	9a: Reading comprehension	Language
	9b: Verbal comprehension	Language
	9c: Reading	Language
	10: Sentence recall	Memory
Factor 3: working memory/memory	2: Examiner’s name (immediate recall)	Memory
	5: Current month	Orientation
	7: City	Orientation
	12: Digit span (series)	Attention
	13: Verbal fluency	Language
	14: Examiner’s name (delayed recall)	Memory
	16 + 18: Using cup (photograph + object)	Praxis
	21 + 23: Using spoon (photograph + object)	Praxis
	25: Immediate recall of objects (cup + spoon)	Memory
	35a: Drawing (circle)	Construction
	35b: Drawing (square)	Construction
	36: Auditory span	Attention
	37: Visual span	Attention
	38: Delayed recall of objects (cup + spoon)	Memory
Factor 4: praxis and social skills	1a: Shake hands	Social skills
	1b: Follow directions	Social skills
	1c: Sit/move to table/pull tray	Social skills
	3: Subject’s name	Orientation
	11a + 11b: Repetition (people spend money + baby)	Language
	39: Orientation to name	Orientating head to name
	40: Language ability (free conversation)	Language
Factor 5: naming	8a + 8b: Descriptive naming (cup + spoon)	Language
	15 + 20: Naming object in photograph (cup + spoon)	Language
	17 + 22: Naming object (cup + spoon)	Language
	19 + 24: Forced-choice naming (cup + spoon)	Language

SIB, Severe Impairment Battery. SIB domain allocation refers to the nine domains of the SIB, to which each of the items have been previously assigned. New subscales relate to the new factors generated from factor analysis of the 40 individual SIB items.

### Efficacy of rivastigmine on the previously defined memory, language and praxis higher-order subscales of the SIB

Using the three subscales derived from a previous analysis [13], significantly reduced decline was seen on the memory (least-squares mean (standard error) change from baseline at Week 26: rivastigmine, -0.40 (0.46); placebo, -1.92 (0.47); *P* = 0.010) and language (rivastigmine, -0.48 (0.76); placebo, -2.40 (0.77); *P* = 0.045) higher-order subscales of the SIB. A trend towards reduced decline, narrowly missing significance, was also observed on the praxis higher-order subscale of the SIB in rivastigmine-treated patients compared with placebo (rivastigmine, -0.33 (0.45); placebo, -1.43 (0.46); *P* = 0.051). Calculated effect sizes showed numerically less decline from baseline at Week 26 with rivastigmine versus placebo on the memory (effect size: 0.26) language (0.27) and praxis (0.24) subscales of the SIB.

### Efficacy of rivastigmine on the newly defined SIB subscales: visual; language; working memory/memory; praxis and social skills; and naming

**Figure 1:**
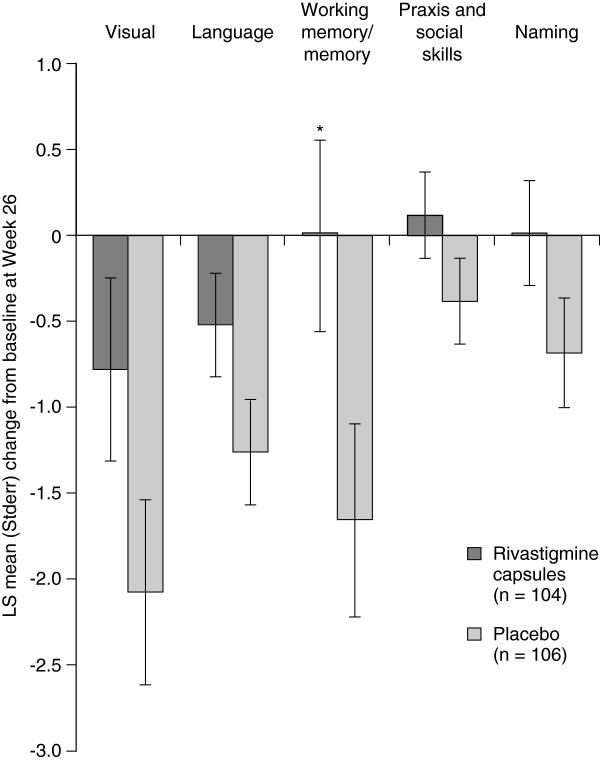
**LS mean change from baseline at Week 26 on the newly defined SIB subscales.** LS, least-squares; Stderr, standard error of the LS mean; SIB, Severe Impairment Battery. Analysis based on the Intent-To-Treat Last Observation Carried Forward (ITT-LOCF) population. *P*-values (derived from LS mean) calculated using an analysis of covariance (ANCOVA) model with treatment and center as factors, and baseline as a covariate. Negative change scores indicate deterioration. Newly defined SIB subscales named Factor 1 = visual, Factor 2 = language, Factor 3 = working memory/memory, Factor 4 = praxis and social skills, Factor 5 = naming. *P < 0.05 versus placebo.

A significant reduction in decline was seen from baseline at Week 26 in rivastigmine-treated patients compared with placebo on the working memory/memory subscale (Factor 3; *P* = 0.018; Figure 1). Despite an apparent trend towards reduced decline in rivastigmine-treated patients compared with placebo on the visual (Factor 1), language (Factor 2), praxis and social skills (Factor 4), and naming (Factor 5) subscales, these differences did not reach significance (*P* = 0.056, *P* = 0.056, *P* = 0.117 and *P* = 0.072, respectively; Figure 1). Based on effect sizes, numerically less decline was observed in patients randomized to rivastigmine compared with placebo on all five newly defined SIB subscales (visual, 0.21; language, 0.22; working memory/memory, 0.28; praxis and social skills 0.12; naming, 0.19). Supporting the ITT-LOCF analysis, the ITT-OC analysis also demonstrated significantly less decline with oral rivastigmine versus placebo on the working memory/memory subscale (*P =* 0.038).

### Efficacy of rivastigmine on the individual items of the SIB

**Figure 2:**
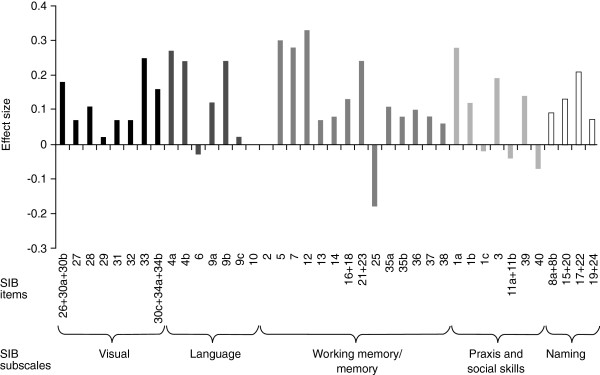
**Effect sizes based on mean change from baseline at Week 26 on individual SIB items.** SIB, Severe Impairment Battery. Analysis based on the Intent-To-Treat Last Observation Carried Forward (ITT-LOCF) population. Negative change scores indicate deterioration.

Calculation of treatment effect sizes for each of the individual items demonstrated efficacy of rivastigmine on a broad range of SIB items, across each of the newly defined SIB subscales (Figure 2). Greatest effect sizes (0.30 and above) were observed on items assessing the current month (item 5; effect size 0.30) and digit span series (item 12; 0.33).

Effect sizes above 0.2 (range 0.21 to 0.28) were observed on these items: shake hands (1a), write name (4a), copy name (4b), city (7), verbal comprehension (9b), naming object (cup + spoon; 17 + 22), using spoon (21 + 23) and shape discrimination (33). Effect sizes of 0.1 and above (range 0.10 to 0.19) were observed on the following items: directions (1b), subject’s name (3), reading comprehension (9a), naming object in photograph (15 + 20), using cup (16 + 18), color naming (26 + 30a + 30b), color block (28), shape identification (30c + 34a + 34b), drawing circle (35a), auditory span (36), and orientation to name (39). For all other items (1c, 2, 6, 8a + 8b, 9c, 10, 11a + 11b, 13, 14, 19 + 24, 25, 27, 29, 31, 32, 35b, 37, 38 and 40) effect sizes were <0.1, and considered similar between rivastigmine and placebo groups, with the exception of item 25 (immediate recall) for which the calculated effect size of −0.18 suggested better performance in the placebo group.

## Discussion

AD is associated with gradual loss of cholinergic transmission in the brain, which manifests as progressive deterioration of cognitive function [14]. As cholinergic deficits correlate with disease stage, treatment strategies that increase levels of acetylcholine available in cholinergic synapses may become more relevant as the disease progresses.

Several clinical studies and retrospective analyses of trial databases have investigated the efficacy of rivastigmine in patients with moderately severe AD. Analysis of a 52-week study of oral rivastigmine investigated the long-term efficacy of rivastigmine in patients with AD stratified by baseline dementia severity, determined using the GDS [15]. Patients with a GDS score ≥5 (moderately severe disease) receiving 1 to 4 mg/day or 6 to 12 mg/day rivastigmine capsules showed significant improvements compared with placebo on the ADAS-cog during an initial 26-week, double-blind treatment phase [15]. At Week 52, following a further 26 weeks of open-label treatment with 6 to 12 mg/day rivastigmine, ADAS-cog performance was significantly superior in treated patients compared with projected placebo [15]. These findings suggest that rivastigmine may have sustained cognitive benefits in patients with moderately severe AD. Furthermore, retrospective, pooled analysis of data from three randomized, placebo-controlled, double-blind, six-month clinical trials of patients with probable AD (MMSE score of 10 to 26) provided evidence for a cognitive benefit of rivastigmine treatment in the subset of patients with more severe AD (MMSE score of 10 to 12) [7]. In support of these findings, further pooled analysis of these studies, in which patients randomized to receive 6 to 12 mg/day rivastigmine capsules were stratified according to dementia severity, reported significant efficacy of rivastigmine versus placebo on the ADAS-cog across all disease stages, including moderately severe AD (MMSE score of ≤ 15) [16].

Although these analyses suggest cognitive benefits of rivastigmine in moderately severe AD, the scales commonly used to assess cognitive efficacy in clinical trials of patients with mild-to-moderately severe AD, for example, the ADAS-cog, may not be sensitive to the full range of cognitive changes in patients with severe AD, owing to floor effects and limited ability of these patients to complete assessments. Tests designed to be sensitive within the capabilities of patients with more advanced stage AD, such as the SIB, may provide further insight into the efficacy of rivastigmine on specific aspects of cognitive function.

A previous randomized, placebo-controlled clinical trial reported significant efficacy of rivastigmine on the total SIB in patients with moderately severe-to-severe AD [9]; SIB data from this trial formed the basis of the current analysis, in which we sought to further investigate the pattern of cognitive efficacy of rivastigmine in patients with severe AD. Rivastigmine treatment was associated with significant efficacy on the higher-order memory and language subscales of the SIB, as defined by a previous analysis of the nine SIB domains [13]. A trend toward numerically greater efficacy was also observed on the praxis subscale.

In the version of the SIB used in this analysis, the nine domains of the SIB comprised 40 items, which assess a diverse range of cognitive impairments, from awareness of the current month, to ability to identify everyday objects, for example, a cup and spoon. Given the broad range of cognitive functions assessed by the items encompassed in the nine SIB domains, we performed a new factor analysis of the SIB using the individual items, as opposed to domains, which allocated the 40 items to one of five factors (two more than the previously published analysis). Rivastigmine demonstrated significant efficacy compared with placebo on the new subscale we termed working memory/memory, based on the range of SIB items encompassed in this subscale. Based on effect sizes, a numerical trend toward greater efficacy was also observed on each of the newly defined subscales in rivastigmine-treated patients compared with placebo. The lack of a significant treatment effect on the visual, language, praxis and social skills, and naming factors may have resulted from floor and ceiling effects on some individual SIB items. Since the majority of studies in AD have been performed in patients of mild-to-moderate disease stage, issues related to these effects in patients with severe AD have not been routinely addressed in clinical studies. In the current analysis, it is possible that floor or ceiling effects at baseline may have influenced loading on to factors, and the ability to observe treatment effects versus placebo.

In addition to investigating efficacy on previously defined and newly defined subscales, we also calculated the magnitude of treatment effects on individual SIB items. Efficacy on these individual items may translate into clinically meaningful benefits for patients and their caregivers. Greatest effect sizes were observed on items assessing current month and memory span (digit series). Both of these items form part of the newly defined working memory/memory subscale and the previously defined memory subscale, and may account in part for the significant efficacy of treatment observed on these subscales. However, overall, rivastigmine was shown to demonstrate efficacy on a wide range of items (based on effect sizes), spanning across both the newly defined and previously defined SIB subscales. These findings suggest that the observed efficacy of rivastigmine on the SIB is not a result of efficacy on a few individual items or a few cognitive domains, but rather is a fairly consistent, cumulative effect across a wide range of cognitive tasks. These findings are encouraging, as they suggest rivastigmine may have broad cognitive efficacy in severe AD.

Limitations of this study include its retrospective nature; this was an exploratory analysis and was only intended to be hypothesis forming. This study was not powered to detect differences on the individual SIB items, previously defined subscales, or on the new subscales derived by factor analysis of the SIB items. These considerations may reduce the robustness of these results. Larger studies are needed to confirm these findings, and to further investigate the cognitive efficacy of oral rivastigmine in patients with severe AD.

Additional information on the pattern of cognitive benefit of rivastigmine in patients with severe AD is provided by the ACTION study (ClinicalTrials.gov identifier NCT00948766) [8]. The SIB was a co-primary outcome measure to assess cognition in this study. The ACTION study demonstrated significantly greater efficacy of 13.3 mg/24 h versus 4.6 mg/24 h rivastigmine patch on cognition, as measured by the change from baseline at Week 24 on the total SIB score, without a marked impact on safety and tolerability [8]. To our knowledge, this was the first clinical trial of the rivastigmine patch in patients with severe AD; primary and *post-hoc* analyses of data obtained in this study will allow investigation into the use and specific cognitive effects of the high dose 13.3 mg/24 h rivastigmine patch in this indication. Furthermore, given that 13.3 mg/24 h patch is now approved for the symptomatic treatment of severe AD in the USA [5], investigating physician experiences with high-dose patch in this indication will provide further information on how trial findings may translate into clinical practice.

## Conclusions

In this *post-hoc* analysis, oral rivastigmine demonstrated efficacy on the previously defined memory and language subscales, newly defined working memory/memory subscales, and several individual items of the SIB in patients with moderately severe-to-severe AD. The cumulative effect observed across a broad range of tasks corroborates previous evidence, and reinforces that rivastigmine may be an effective therapy in the treatment of severe AD.

## Abbreviations

ACTION: *ACT*ivities of daily living and cognit*ION*; AD: Alzheimer’s disease; ADAS-cog: Alzheimer’s Disease Assessment Scale-cognitive subscale; ADCS-ADL: Alzheimer’s Disease Cooperative Study-Activities of Daily Living; ADCS-CGIC: Alzheimer’s Disease Cooperative Study-Clinical Global Impression of Change; ADL: Activities of daily living; ANCOVA: Analysis of covariance; GDS: Global Deterioration Scale; ITT-LOCF: Intent-To-Treat Last Observation Carried Forward; ITT-OC: Intent-To-Treat Observed Case; LS: Least-squares; MMSE: Mini-Mental State Examination; NPI-10: 10-item Neuropsychiatric Inventory; NPI-4: 4-item Neuropsychiatric Inventory; SD: Standard deviation; SIB: Severe Impairment Battery; Stderr: Standard error of the LS mean.

## Competing interests

SF has served as a scientific consultant to companies marketing, developing or contributing to the development of treatments for cognition, including Accera, Baxter, Bristol-Myers Squibb, Cebria, Dart Neuroscience, Eisai, Janssen AI, Eli Lilly, Lundbeck, Lupin, MedAvante, Merck, Neuronix, Neurotrack, Targacept and United Biosource. There was no payment from Novartis for participation in this article. His institution has received grant/contract support for clinical trials from Accera, Dart Neuroscience, Eisai, Eli Lilly, EnVivo, GE Healthcare, Genentech, Janssen, Janssen AI, Lundbeck, Merck, Roche, Neuronix, Takeda and Bristol-Myers Squibb. He also has stock options from Accera, Intellect Neurosciences and MedAvante, and stock in Lexicon Pharmaceuticals. SK has no relevant conflicts of interest to declare. MS and XM are full-time employees and stock holders of Novartis Pharmaceuticals Corporation, East Hanover, NJ, USA. Novartis developed and manufactures rivastigmine, and sponsored the large, multicenter, randomized, double-blind trials that led to the approval of rivastigmine in the USA for mild-to-moderate and severe AD. Novartis also sponsored the study of oral rivastigmine in moderately severe-to-severe AD which forms the basis of the current analysis.

## Authors’ contributions

SF, SK, MS and XM made substantial contributions to the analysis and interpretation of the data, were involved in drafting the manuscript or revising it critically for important intellectual content and have given final approval of the version to be published.
